# Expression of Bruton´s tyrosine kinase in different type of brain lesions of multiple sclerosis patients and during experimental demyelination

**DOI:** 10.3389/fimmu.2023.1264128

**Published:** 2023-11-13

**Authors:** Maria L. Elkjaer, Mie R. Waede, Christina Kingo, Karina Damsbo, Zsolt Illes

**Affiliations:** ^1^ Department of Neurology, Odense University Hospital, Odense, Denmark; ^2^ Department of Clinical Research, University of Southern Denmark, Odense, Denmark; ^3^ Department of Molecular Medicine, University of Southern Denmark, Odense, Denmark; ^4^ BRIDGE – Brain Research Interdisciplinary Guided Ecxellence, University of Southern Denmark, Odense, Denmark

**Keywords:** smoldering MS, Bruton´s tyrosine kinase, white matter, brain lesions, microglia, B cells, cuprizone, BTKi

## Abstract

**Background:**

Inhibition of Bruton’s tyrosine kinase (BTK) is an emerging multiple sclerosis (MS) therapy. BTK inhibitors (BTKi) cross the blood-brain barrier and modulate B cells and microglia, major cellular players in active and chronic active lesions.

**Objective:**

To assess potential lesional and cellular targets of BTKi, we examined BTK expression in different type of MS white matter (WM) lesions, in unmanipulated CNS resident cells, and in a degenerative MS model associated with microglia activation *in vivo*.

**Methods:**

We examined BTK expression by next-generation RNA-sequencing in postmortem 25 control WM, 19 NAWM, 6 remyelinating, 18 active, 13 inactive and 17 chronic active lesions. Presence of B cells and microglia were examined by immunohistochemistry. CNS resident cells were isolated from the mouse brain by magnetic sorting. BTK expression was examined by quantitative PCR in isolated cells and dissected corpus callosum from mice treated with cuprizone (CPZ).

**Results:**

BTK expression was significantly increased in active and chronic active lesions with upregulated complement receptors and Fcγ receptors. Active lesions contained high number of perivascular B cells, microglia, and macrophages. Chronic active lesions were characterized by microglia/macrophages in the rim. Microglia expressed BTK at high level (120-fold) in contrast to other CNS cell types (2-4-fold). BTK expression was increasing during CPZ treatment reaching significance after stopping CPZ.

**Conclusion:**

Considering BTK expression in MS lesions and resident cells, BTKi may exert effect on B cells, microglia/macrophages in active lesions, and limit microglia activation in chronic active lesions, where tissue damage propagates.

## 1 Introduction

The signaling molecule Bruton’s tyrosine kinase (BTK) is an intracellular enzyme produced by B cells and most hematopoietic cells. It mediates signals important in B cell differentiation, activation, proliferation, and antigen presentation (1–3). BTK produced by B cells is also involved in integrin adhesion, migration of B cells, and toll-like receptor (TLR)-mediated proinflammatory cytokine production (4–6). BTK also plays a role in innate immune responses. It mediates phagocytosis and cytokine secretion through FcγR of cells of hematopoietic origin including macrophages (1, 7–9). Additionally, BTK protein is expressed by the CNS resident cells microglia and at low levels in subtypes of astrocytes in experimental models (9–11).

Inhibition of BTK has been an emerging treatment option in multiple sclerosis (MS) considering the role of B cells and microglia in pathways contributing to both acute inflammation and chronic disease worsening (12–14). In contrast to anti-CD20-mediated B cell depletion, BTK inhibitors (BTKi) modulate B cell function without causing long-term and broad B cell depletion that may raise safety concerns.

BTKi are divided into different subtypes depending on the binding sites and mode of inhibition. Irreversible covalent binding allows for lower, less frequent drug doses due to increased affinity and prolonged action. However, it could potentially enhance immunogenicity (9, 15–17).

So far, five BTKi are under clinical evaluation for both relapsing and progressive MS: evobrutinib, tolebrutinib, remibrutinib and orelabrutinib with irreversible covalent binding, and fenebrutinib with reversible non-covalent binding (9, 15–17). The pharmacokinetics and pharmacodynamics of these BTKi vary. CNS penetrance may also differ, although data are scarce (9). In the cerebrospinal fluid (CSF), the concentration of evobrutinib 2-3 hours post-administration is lower than its half-maximal inhibitory concentration, whereas the concentration of tolebrutinib is higher, suggesting superior CNS penetration compared to evobrutinib as well as fenebrutinib (9).

Published phase II clinical trial data for evobrutinib and tolebrutinib have shown promising results. In the double-blind trial of evobrutinib, 267 patients with relapsing MS were treated with three different doses of evobrutinib compared to placebo, or open-label dimethyl fumarate for 24 weeks. The number of gadolinium (Gd)-enhancing lesions and annualized relapses rate (ARR) were decreased and remained low during the open-label extension period (9, 18). Neurofilament levels in the serum were reduced and maintained up to week 144 (9, 17). The volume of slowly expanding lesions on the MRI was also reduced (17). Two identical phase III clinical studies are comparing ARR between oral evobrutinib 75 mg twice daily to teriflunomide in relapsing MS (EVOLUTION 1 and 2) (15–17). In a randomized, placebo-controlled Phase IIb trial with 130 adults, four doses of oral tolebrutinib over 12 weeks were compared to 4-week placebo run-in and run-out in a 16-week cross-over design. The number of Gd-enhancing and new or enlarging T2 lesions were decreased compared to the placebo period, and no new Gd-enhancing lesions were observed after 12 weeks of treatment (19). Similar efficacy was observed in the highly active MS subpopulation (9). In the long-term extension study, ARR remained low at 72 weeks, and no increase in the EDSS was observed (9, 17). Two identical ongoing phase III studies (GEMINI 1 and 2) are evaluating ARR compared to teriflunomide for approximately 36 months in people with relapsing MS. Phase III studies in inactive secondary progressive MS and primary progressive MS (HERCULES and PERSEUS) examine the effect of tolebrutinib on 6-month confirmed disability progression up to approximately 48 months compared to placebo (9, 15–17).

Three phase III trials have been initiated with oral fenebrutinib: two against teriflunomide in relapsing MS (FENhance and FENopta) and one in primary progressive MS (PPMS) against placebo compared to ocreluzimab *versus* placebo (FENtrepid). The primary outcome in the relapsing MS trial is ARR over minimum 96 weeks, and in the PPMS study time to onset of composite 12-week confirmed disability progression. Both studies enrol several hundred patients (15, 17). Two identical phase III clinical studies compare ARR between remibrutinib in relapsing MS against teriflunomide in several hundred patients. The double-blind double-dummy phase will be followed by open label extension for up to five years (15–17). Three doses of orelabrutinib are compared to placebo in a phase II study in relapsing MS, with the primary outcome being the number of Gd-enhancing lesions. After 24 weeks, the patients will be eligible to enter an open label extension phase (15, 17).

Beside targeting both adaptive and innate peripheral immune responses, the CNS penetrance and effect on both B cells and microglia/macrophages suggest that BTKi can be an attractive approach to treat low-level inflammation in smouldering MS. Activation and functional change of microglia in the normal-appearing white matter (NAWM) and in the rim of chronic active lesions participate in propagation of chronic tissue damage and maintaining such low-level chronic inflammation in concert with astrocytes, B and T lymphocytes (20–22). Microglia activation in the NAWM can be visualized by PET, while chronic active lesions are reflected on MRI as slowly evolving (SEL) and paramagnetic rim lesions (PRLs) (14, 23–25). Chronic active lesions as PRLs are already present in the early stage of MS and associate with progressive MS and advanced clinical disability (26–29). Additional pathological features behind smouldering MS maybe also targeted by BTKi, including cortical lesions induced by microglia activation and T-B cell interactions in meningeal follicle-like structures (20).

To address which WM lesions can be potentially targeted by BTKi, we compared BTK expression in 73 different WM lesions in postmortem brain samples from progressive MS: NAWM, active lesions, chronic active lesions, inactive lesions and remyelinating lesions. We also compared BTK expression in CNS resident cells to explore potential effect of BTKi on other cell types. Finally, we examined expression of BTK during cuprizone (CPZ)-induced demyelination associated with microglia activation *in vivo*.

## 2 Materials and methods

### 2.1 Brain samples and RNA sequencing

Brain tissue samples for this study, including control white matter (n=25), normal-appearing white matter, NAWM (n=20), remyelinating (n=6), active (n=17), inactive (n=14), and chronic active lesions (n=17), were supplied by the UK Multiple Sclerosis Tissue Bank (UK Multicentre Research Ethics Committee, MREC/02/2/39), funded by the Multiple Sclerosis Society of Great Britain and Northern Ireland (registered charity 207,495) (**Table 1**). RNA sequencing data generated from these tissues and published previously (21) were used to examine expression of BTK. Raw data are available in GEO (ID GSE138614). In short, lesion types were defined as described (30) based on the morphology and abundance of MHC II (HLA DR^+^)-expressing cells (Thermo Scientific MA1-25914) as well as degree of myelin evaluated by staining for myelin oligodendrocyte glycoprotein (MOG,1:20, mouse, monoclonal R. Reynolds, in-house, Imperial College, UK) followed by biotinylated secondary antibody (Vector Laboratories, BA-1100), avidin/biotin staining (Vector Laboratories, Burlingame, CA) and DAB staining (Vector Laboratories, Burlingame, CA) (21). B cells were identified by using ant-CD20 immunostaining (Abcam ab78237). All subsequent experimental and bioinformatic analyses, encompassing microdissection, total RNA isolation, library preparation, 2x80 bp paired-end RNA sequencing on an Illumina NextSeq550, data preprocessing (demultiplexing, filtering, genome alignment, and counting), raw data analysis and quality control, have been previously described (21). Data preprocessing was done as detailed in (22). A generalized linear model, adjusted for library size and biological replicates (same lesion type, same sample, from same patient) and corrected for age and sex of the patients, were used to identify significant differences in BTK expression. Differences were determined based on adjusted P value filtering using the Benjamini and Hochberg procedure (false discovery rate, FDR) between MS WM brain areas and control WM brain areas (21, 22).

**Table 1:** Clinical and demographic data.

MS patients				Controls	
Sex (f/m)	Age of death (years)	Disease duration (years)	Time in progressive phase (years)	Sex (f/m)	Age of death (years)
M	39	10	2	M	35
F	54	30	21	M	68
F	61/62	50	19	M	68
M	51	18	14	F	50
F	45	25	3 to 11	F	61
M	42	21	15		
M	50	29	20		
M	75	31	Primary progressive		
F	53	27	–		
F	54	26	15		

**M**, male; F, female.

### 2.2 Isolation of CNS resident cells from mouse brain

Single cell suspensions of 3-5 brains from postnatal (P4-8) C57BL/6J mice were prepared by using SuperMACS II Separator (MACS Miltenyi Biotec, Lund, Sweeden, Catalogue 130-044-104) and labeled with CD11b Microbead Kit (microglia, MACS Miltenyi Biotec, Lund, Sweeden, Catalogue 130-094-634), ACSA-2 Microbead Kit (astrocytes, MACS Miltenyi Biotec, Lund, Sweeden, Catalogue 130-097-678), CD140a/PDGFRA Microbead Kit (oligodendrocyte precursor cells, OPC, MACS Miltenyi Biotec, Lund, Sweeden, Catalogue 130-097-678), O4 Microbead Kits (premature oligodendrocytes, MACS Miltenyi Biotec, Lund, Sweeden, Catalogue 130-094-543) or Neuron Isolation Kit (MACS Miltenyi Biotec, Lund, Sweeden, Catalogue 130-098-752) followed by positive selection (glial cells) or negative selection (neurons) by a SuperMACS™ II separator. The purity of the cell fractions was between 74-89% for glia cells and 52% for neurons.

### 2.3 Cuprizone model and microglia staining

C56BL/6 female mice, aged 8-9 weeks (Taconic Ltd., Ry, Denmark) were either fed with powdered standard chow mixed with 0.2% CPZ (Sigma Aldrich, MO, USA) and) for 6 weeks or for 6 weeks followed by 2 weeks of normal chow. An age-matched control group was maintained on normal diet for comparative analysis. Brains were collected from anesthetized and PBS-perfused mice, and snap frozen in isopentane chilled with liquid nitrogen. Mice were bred according to protocols approved by the Danish Animal Health Care Committee (2014-15-00369) and EU Directive 2010/63/EU for animal experiments. Snap-frozen mouse brains were cryosectioned to a thickness of 35 μm. The slides were fixed with 4% PFA for 15 min. Antigen retrieval was done for 30 min in citrate buffer (10 mM tri-sodium citrate, 0.05% tween 20) pH 6 using a steam cooker. Peroxidase activity was removed by treading the slides with 0.3% H_2_O_2_ for 10 min. To detect microglial cells, slides were washed three times with TBS-T (TBS with 0.1% Triton-X 100), blocked 30 min in 10% bovine serum (NBS), and incubated O/N with 1:500 pAb anti Iba (Applied biosystem, AB178846). Following another TBS-T wash, they were treated for 1 hour with secondary antibody (Goat anti Rabbit biotin Sigma SAB4600007 1:4000). After washing, slides were incubated 30 min with Avidin-biotin horseradish peroxidase complex (Vector Laboratories, CAT. PK-4000) and developed using the DAB substrate kit (SK-4100 DAB Substrate Kit, Vector Laboratories).

### 2.4 Quantitative PCR (qPCR)

RNA was extracted with RNeasy Micro kit (QIAGEN, Catalogue 217084) and cDNA were synthesized with The High Capacity cDNA Reverse Transcription Kit (Life Technologies, Catalogue 4366596). BTK primers were obtained from BioRad (qMmuCID0023085). HPRT1 and GAPDH primers were obtained from Invitrogen (#933319 C1010, #933319 C1180, #855449 R5552 and #855449 R5552). All primers were SYBR Green based.

### 2.5 Statistical analysis

The method for identifying differentially expressed genes in different lesion types *vs.* control WM is as previously described (21, 22). In short, differentially expressed genes between lesion types compared to control WM were identified using generalized linear models adjusted for lesion distribution, age, and sex, followed by P value filtering (Benjamini-Hochberg). Expression level of BTK between the different brain cells was analyzed by One-Way ANOVA corrected for multiple comparisons using the statistical hypothesis test “Tukey”. BTK expression during CPZ feeding and after cessation of CPZ was compared to control using an unpaired two-tailed t-test. Statistical significance was set at FDR (false discovery rate) or p< 0.05. Spearman correlation was used to examine expression among BTK and other genes in the MS brain tissue.

## 3 Results

### 3.1 Increased BTK expression in active and chronic active lesions

**Figure 1:**
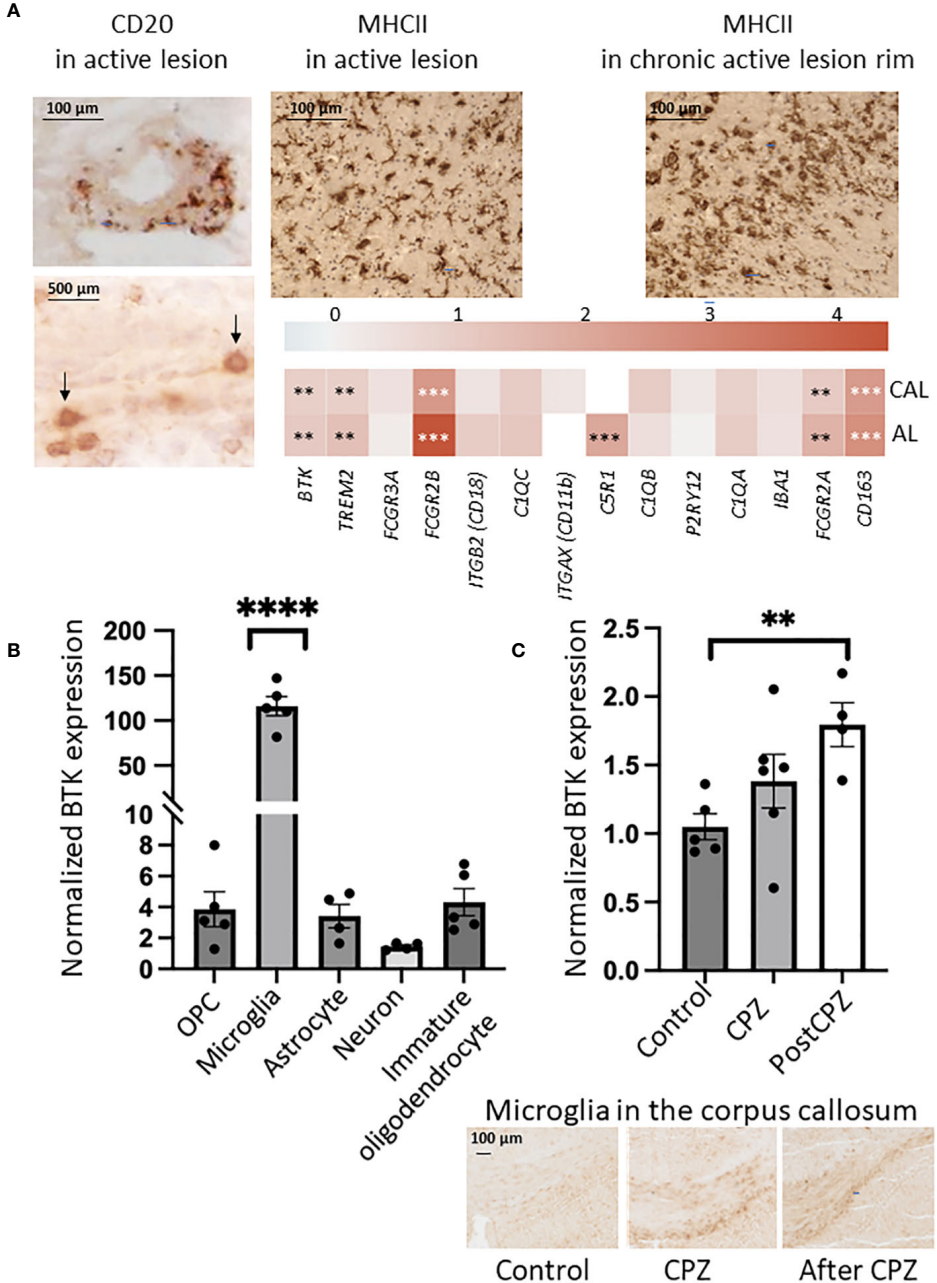
**(A)** Immunohistochemistry indicated the presence of B cells (CD20, brown and arrows) and microglia/macrophages (MHCII) in the active lesion, and microglia/macrophages (MHCII, brown) in the rim of chronic active lesion. Expression of microglia genes (logFC) is shown in a heatmap in active (AL) and chronic active lesions (CAL): FDR less than 0.01 is marked with two stars (**), FDR less than 0.001 is marked with three stars (***). **(B)** BTK expression examined by quantitative PCR was increased in microglia isolated from postnatal mouse brain, while expression in other resident cells was minor. OPC: oligodendrocyte precursor cells. One-Way ANOVA corrected for multiple comparisons using the statistical hypothesis test “Tukey”. ****p<0.0001. **(C)** BTK expression examined by quantitative PCR was increasing during 6-week cuprizone (CPZ) treatment and 2 weeks after CPZ was stopped. Unpaired two-tailed t-test. **p<0.01. Presence of microglia in the corpus callosum during the three conditions was examined by Iba1 immunostaining.

To explore BTK expression in the MS brain and compare expression among different lesion types, we microdissected 73 brain tissues from 10 patients with progressive MS. The MS tissues covered NAWM and WM lesion types: active, chronic active, inactive, and remyelinating lesions. Additionally, 25 control WM tissues were obtained from 5 individuals without neurological diseases (**Table 1**). Following next-generation RNA sequencing, BTK expression in the lesion types was compared with BTK expression in the control WM. BTK expression was significantly increased in active and chronic active lesions (FDR<0.05) (**Table 2**). Using CD20 and MHCII/HLA-DR immunohistochemistry, we examined the presence of B cells and microglia/macrophages in these lesions. Active lesions contained a high number of perivascular B cells beside microglia/macrophages, while chronic active lesions were characterized by the presence of microglia in the rim (**Figure 1A**). We also examined the expression of several microglia genes in these lesion types and their correlation with BTK expression (**Figure 1A**). We found no upregulation of the homeostatic microglia gene of P2RY12 (LogFC and FDR 0.054 and 0.96 in active and 0.29 and 0.46 in chronic active lesions, respectively) indicating that the increased number of microglia in the chronic active lesion rim and in active lesions may not represent a resting phenotype. Therefore, we examined expression of genes related to activation of microglia and their correlation with BTK expression. Several microglia genes were upregulated in active and chronic active lesions along BTK gene compared to control white matter, including *TREM2* (active: logFC: 1.4 FDR: 0.01; chronic active: logFC 0.9, FDR: 0.02), *FCGR2B* (active: logFC 4.4, FDR<0.001; chronic active: logFC 2.5, FDR <0.001), *C5R1* (active: logFC 2.0, FDR<0.01), *FCGR2A* (active: logFC: 1.7, FDR <0.01; chronic active: logFC 1.1, FDR=0.02), and *CD163* (active: logFC 2.7 FDR <0.001; chronic active: logFC 2.4, FDR<0.001) (**Figure 1A**, heatmap). The expression of BTK gene also correlated with the expression of *TREM2* (p<0.01), *C1QA* (p<0.01), and *C1QC* (p<0.01) across lesion types. In contrast to microglia genes, CD20 was not upregulated in the lesions.

**Table 2:** Expression of BTK in different white matter lesions in multiple sclerosis.

Brain lesion type	Log_2_FC	FDR
NAWM *vs* WM	0.90	0.13
Inactive *vs* WM	0.38	0.50
Remyelinating *vs* WM	0.84	0.30
Active *vs* WM	1.13	0.03
Chronic active *vs* WM	1.00	0.02

NAWM (normal-appearing white matter) n=19; WM (control white matter) n=25; Inactive lesion, n=13; Remyelinating lesion, n=6; Active lesion, n=18; chronic active lesion, n=17. The generalized linear model adjusted for library size and biological replicates (same lesion type//same sample//from same patient) and corrected for age and sex of the patients was used to define significant difference in BTK expression calculated by adjusted P value filtering using the procedure of Benjamini and Hochberg (false discovery rate, FDR) between MS brain areas and control brain areas was.

### 3.2 BTK is primarily expressed in microglia within the CNS

To compare BTK expression among different CNS resident cell types, we isolated microglia, astrocytes, oligodendrocyte precursor cells (OPCs), premature oligodendrocytes, and neurons from brains of postnatal mice. BTK expression was examined using qPCR and normalized to housekeeping genes. Microglia displayed a 120-fold significant increase in BTK expression compared to a only 2-4-fold increase observed in the other cell types relative to the internal control genes (**Figure 1B**).

### 3.3 Increased BTK expression in the mouse brain after feeding with cuprizone

Next, we examined if BTK expression is increased in the degenerative model of MS characterized by microglia activation *in vivo*. Mice were fed with cuprizone (CPZ) for 6 weeks. The corpus callosum was microdissected from mice after 6-week CPZ feeding (n=6), 2 weeks after cessation of CPZ (n=4) and from control mice without CPZ (n=5). BTK quantified by qPCR showed increased expression during CPZ treatment and was significantly elevated after CPZ treatment (**Figure 1C**).

## 4 Discussion

BTK inhibition is an emerging treatment of multiple sclerosis based on completed and ongoing phase II and III clinical trials (9, 15–17). Given that BTKi can cross the blood-brain barrier (BBB) and modulate both B cell and microglia/macrophage functions (9), it potentially allows direct manipulation of pathological drivers and mechanisms of MS within the CNS. Therefore, we examined which MS lesion types have increased BTK expression, the target of BTKi. We also determined which CNS cell types express BTK to identify potential cellular targets of BTKi. Finally, we explored if activation of microglia *in vivo* results in increased expression of BTK.

Our findings show a significant increase in BTK expression in two types of MS lesions: active and chronic active lesions. Such increased expression may be related to the presence of activated microglia in the rim of chronic active lesions, and to the presence of B cells and microglia/macrophages in the active lesions. While expression of the homeostatic microglia gene P2ry12 was not increased in these lesions, we found increased expression of TREM2, several Fcγ receptors, and complement receptors that trigger inflammatory, cytolytic, and phagocytic activities (31). These molecular changes indicated the presence of proliferating and phagocytosing microglia in the active and chronic active lesions with significantly increased BTK expression. While anti-CD20 immunostaining indicated the presence of B cells in both lesion types, we were unable to capture the CD20 gene expression in the tissue transcript pool. This may be related to the lower number of B cells. Indirectly, this may emphasize microglia as the primary target of BTKi in these CNS lesions.

Chronic active lesions in the WM are considered as one of the main drivers of smoldering MS (20). These lesions are characterized by microglial and astrocytic activation at the rims where tissue damage slowly expands (20–23, 32). Unpublished data also indicate increased BTK expression around the rim of lesions in autopsy samples from patients with SPMS (9). In our study, chronic active lesions with significantly increased BTK expression had the highest number of uniquely differentially expressed genes (unique DEGs over 2000) compared to the other lesion types and were also the most different from remyelinating lesions (21). Specific pathways based on these DEGs were also enriched in these lesions, and *de novo* network enrichment indicated neuron/synaptic gene activity indicating intrinsic irreversible pathogenic events (21). Recent findings from single-nucleus RNA sequencing combined with postmortem ultra-high-field 7T MRI indicate a coordinated interplay among T and B lymphocytes, microglia and astrocytes creating a proinflammatory environment in the rim of chronic active lesions (23). An increasing amount of research explores the MRI and PET correlates of chronic active lesions. Findings suggest that lesions with iron deposition in the rim, termed paramagnetic rim lesions (PRLs), and slowly evolving lesions (SELs) likely represent the histological entity (24–29, 32, 33). PRLs are present in the very early phase of MS, at the first clinical sign or even in prodrome and their number correlates with cognitive deficits and worsening disability (26–29). Serum and/or CSF levels of emerging biomarkers, such as neurofilament light chain (NFL), glial fibrillary acidic protein (GFAP), and chitinase 3-like 1 (CHI3L1) correlate with the number of PRLs (34–36). Interestingly, in our study, the chronic active lesions with increased BTK expression showed the highest expression of CHI3L1 compared to the other lesion types, and its expression was localized to the rim (36).

We compared the gene expression of BTK in CNS resident cells isolated from the postnatal mouse brains and observed the highest expression in microglia. Other cell types, such as astrocytes, oligodendrocyte precursor cells (OPC), immature oligodendrocytes, and neurons exhibited lower levels of expression; however, the purity of the isolated neurons was notably low (47.34%). A previous study has shown BTK protein expression in neurons in the mouse brain and in a human neuroblastoma cell line (37). Moreover, BTK protein expression was present in S100b^+^ astrocytes of mouse cerebellar organotypic slices but not in rat GFAP^+^ astrocytes in the spinal cord (10, 11). Collectively, these findings indicate that microglia are the main source of BTK in the CNS at both gene and protein levels. BTKi can inhibit the microglia proliferation and activation in mouse models, both *in vitro* and *in vivo*. Furthermore, in human induced pluripotent stem cell-derived microglia cultures, BTKi modulated the gene expression and proinflammatory cytokine production of microglia (9, 38).

We employed the CPZ model to investigate the expression of BTK in an experimental system associated with *in vivo* microglia activation. During CPZ-induced demyelination in the corpus callosum, there is increased microglia infiltration. In our prior work, we showed changes in the microglial proteome and differential expression of microglia genes with additional upregulation even after CPZ was stopped, during the remyelinating phase (36, 39–42). In contrast to the autoimmune animal model of MS (experimental autoimmune encephalomyelitis), the CPZ model displays minimal adaptive immune responses, allowing for a more accurate estimation of BTK expression linked to microglia. Our results revealed an increased BTK expression during the CPZ treatment, and an even higher increase post-treatment. The post-treatment phase aligns with remyelination, indicating consistent upregulation of BTK expression throughout both de- and remyelination in the model. As B cells do not infiltrate demyelinating lesions in the CPZ model in contrast to microglia and astrocytes, and we found low-level expression of BTK in the other CNS resident cells including astrocytes, elevated BTK expression is most likely related to the microglia activation or the increased number of microglia cells in the corpus callosum. In cerebellar organotypic slice culture, BTK was observed in microglia (73.1%), and S100b^+^ astrocytes (25.6%) but not in oligodendrocytes or OPCs during lysophosphatidylcholine (LPC)-induced demyelination (10). Although the remyelination phase in our CPZ-model showed increased BTK expression, a direct negative effect of BTKi on remyelination seems unlikely, given the absent protein and low-level gene expression of BTK in oligodendrocytes and OPCs. BTK expression was not increased in remyelinating MS lesions either and the gene expression profile of these remyelinating lesions was distinctly different from chronic active lesions that showed increased BTK expression (21). Indeed, when demyelinated cerebellar slices were treated with BTKi, there was a notable enhancement in remyelination, both in tadpoles and in a cortical demyelination model. This treatment not only reduced microglial migration to demyelination sites but also inhibited the loss of oligodendrocytes (9, 10).

In summary, our findings demonstrate that BTK gene expression in the MS brain is significantly increased in two types of lesions, the active and chronic active lesions, both characterized by presence of B cells and microglia that are cellular targets of BTKi. We also observed the highest expression of BTK in microglia isolated from the unmanipulated postnatal mouse brain and in the CPZ disease model characterized by activation of microglia *in vivo*. These results indicate that BTKi, capable of crossing the BBB, may impact the evolution and expansion of both the active and chronic active lesions associated with smouldering MS. As a caveat, increased gene and protein expression of BTK may not necessarily mean increased activation, because BTK is activated by phosphorylation. Nevertheless, the therapeutic effect of silencing BTK by RNA interference in cancers with upregulated BTK highlights the potential that targeting increased expression may be beneficial (41). Therefore, inhibiting upregulated BTK in B cells and microglia may restrict both active and smoldering MS and complement BTK inhibition in the peripheral compartment.

## Data availability statement

Publicly available datasets were analyzed in this study. This data can be found here: Raw sequence are freely available at GEO (access number: GSE138614).

## Ethics statement

The studies involving humans were approved by UK Multiple Sclerosis Tissue Bank (UK Multicentre Research Ethics Committee, MREC/02/2/39. The studies were conducted in accordance with the local legislation and institutional requirements. The participants provided their written informed consent to participate in this study. The animal study was approved by Danish Animal Health Care Committee (2014-15-00369). The study was conducted in accordance with the local legislation and institutional requirements.

## Author contributions

MLE: Data curation, Formal Analysis, Funding acquisition, Investigation, Methodology, Supervision, Validation, Writing – review & editing. MW: Data curation, Formal Analysis, Investigation, Methodology, Writing – review & editing. CK: Data curation, Investigation, Methodology, Writing – review & editing. KD: Data curation, Formal Analysis, Investigation, Methodology, Writing – review & editing. ZI: Conceptualization, Formal Analysis, Funding acquisition, Methodology, Project administration, Resources, Supervision, Validation, Writing – original draft, Writing – review & editing.
